# Effects of 12-week supplementation of *Citrus bergamia* extracts-based formulation CitriCholess on cholesterol and body weight in older adults with dyslipidemia: a randomized, double-blind, placebo-controlled trial

**DOI:** 10.1186/s12944-017-0640-1

**Published:** 2017-12-22

**Authors:** Yong Cai, Guoqiang Xing, Tian Shen, Shuxian Zhang, Jianyu Rao, Rong Shi

**Affiliations:** 1 School of Public Health, Shanghai Jiao Tong University, School of Medicine, Shanghai, 200025 People’s Republic of China; 2 Department of Radiology & Imaging, Institute of Rehabilitation and Development of Brain Function, The Second Clinical Institute of North Sichuan Medical College, Nanchong Central Hospital, Nanchong, 637000 People’s Republic of China; 3 Lotus Biotech.com LLC, John Hopkins University-MCC, 9601 Medical Center Drive, Rockville, MD 20850 USA; 4 Department of Pathology and Laboratory Medicine, David Geffen School of Medicine, University of California at Los Angeles, Los Angeles, CA 90095 USA; 5 School of Public Health, Shanghai University of T.C.M, Shanghai, 201203 People’s Republic of China

**Keywords:** *Citrus bergamia* Extracts, Body mass index, Blood cholesterol, Old adults

## Abstract

**Backgrounds:**

Recent experiments suggest that *Citrus bergamia* extracts could benefit people with dyslipidemia and obesity but this needs to be further validated.

**Methods:**

A total of 98 people age-matched older adults (65 years) with elevated blood lipids were enrolled to receive 12-week supplementation of a *Citrus bergamia* extracts-based formulation (CitriCholess)(*n* = 48) and placebo (*n* = 50).

**Results:**

No group differences were found in baseline bodyweight, body mass index (BMI), blood cholesterol (TC), triglycerides (TG), low density lipoprotein cholesterol (LDL-C) and glucose levels. CitriCholess supplementation resulted in lower levels than placebo in TG (1.83 ± 0.92 vs. 1.95 ± 1.34 mmol/L, *P* = 0.612), TC (5.14 ± 0.98 vs. 5.44 ± 0.77 mmol/L, *P* = 0.097), and LDL-C (3.13 ± 0.74 vs. 3.43 ± 0.62 mmol/L, *P* = 0.032). Compared to placebo, CitriCholess also resulted in greater reductions in body weight (−0.604 ± 0.939 vs. 0.06 ± 0.74 kg, *P* < 0.01), waist circumferences (−0.60 ± 1.349 cm vs. -0.16 ± 1.503 cm, P < 0.01) and BMI (−0.207 ± 0.357 vs. 0.025 ± 0.274, P < 0.01). Additionally, females had a significantly higher level of HDL-C than males. TC was significantly correlated with LDL-C, and to a less degree, with TG. TG was inversely correlated with HDL-C. Body weight and waist circumference were negatively correlated with HDL-C and positively correlated with glucose.

**Conclusion:**

12-week supplementation of CitriCholess could benefit lipid metabolism and weight management in old adults with dyslipidemia.

## Background

Overweight, increased blood concentrations of low-density lipoprotein cholesterol (LDL-C), total cholesterol (TC) and triglycerides (TG) but low levels of high-density lipoprotein cholesterol (HDL-C) are risk factors for atherosclerosis and coronary artery conditions that are common in people with abnormal levels of blood glucose and high blood pressure [1–4]. According to Centers for Disease Control and Prevention (CDC), more than 50% of Americans adults are overweight (with BMI > 25) and more than 20% of Americans are living with obesity (BMI > 30).

LDL-C are known to be directly and independently associated to cardiovascular conditions (CVD) [5] and LDL-C-induced reactive oxygen species (ROS) and increased NADPH oxidase activity are major causative factors in endothelial perturbation and in the pathogenesis of atherosclerosis [6].

Of the available therapies, statins (3-hydroxy- 3-methylglutaryl Co-enzyme A (HMG-CoA) reductase inhibitors) are the most common drugs used for normalizing high blood cholesterol levels, triglycerides and LDL-C [7–12]. Statins inhibit the activity of HMG-CoA reductase which catalyzes the rate-limiting step in mevalonate biosynthesis, a key intermediate in cholesterol metabolism. Statins act to reduce total cholesterol production and switch the LDL-C fraction to HDL-C fraction that contribute to the significant reductions in the incident, morbidity and mortality of CVD [13].

Despite the significant benefits of statins however, many people, especially those with high blood sugar/glucose and high blood pressure, often could not achieve the desired normal levels of TC, TG, LDL-C and HDL-C with statin monotherapy alone. Moreover, more than 40% of people eligible for statins are forbidden from the use of this therapy due to the side effects of statins including muscle discomfort, muscular weakness, or liver conditions and the breakdown of damaged skeletal muscle [14–16]. Statin therapy was associated with elevated blood lactate/ pyruvate ratio suggestive of mitochondrial issues [17]. Thus, alternative approaches are needed for the management of abnormal levels of cholesterol.

Recent studies have shown that *Citrus bergamia* (known as Bergamot) juice and its flavonoids was able to reduce serum levels of lipids and ameliorate the thickening of the arteries through modulating enzymatic activities, anti-oxidation, anti-inflammatory mechanisms and inhibition of monocyte activation and proliferation [18–25]. Bergamot (*Citrus bergamia*) juice-derived flavonoids (CBF) contains about 28–30% of flavonoids and has a unique profile of bioflavonoid and glycosides including neoeriocitrin, neohesperidin, naringin, rutin, neodesmin, rhoifolin and poncirin [26–30]. Preclinical and clinical studies indicate the cholesterol-lowering property of *C. bergamia* flavonoids (CBF) [31–33].

CBF inhibit LDL oxidation and the activity of HMG-CoA reductase [34], and exert therapeutic effects in animal models of pathological arteries [35], as well as significant and sustained hypolipidemic effects and vasoprotective effects in animals models of high blood cholesterol, renal damage, and ischemic stress-induced injury [34, 36–38]. However, so far, few studies have evaluated the effect of CBF in old adults with dyslipidemia.

Waist circumference and fasting plasma triglyceride concentrations have been used as the simple screening tools for the identification of people at risk of atherosclerosis and metabolic syndrome [39–43]. Modification of these risk factors include BMI and waist circumference earlier can help to prevent or delay onset of comorbid conditions [4].

The objective of this exploratory, randomized, placebo-controlled follow-up observation study was to evaluate the safety and effectiveness of 12-week dietary supplementation of CitriCholess, on cholesterol levels in older adults with blood pressure and blood sugar issues.

## Methods

### Samples size calculation and participants recruitment

Sample size calculation was obtained by the calculation of the estimated experimental study parameters. Triglycerides was chosen as the main outcome, assuming a baseline triglyceride concentration at about 2 mmol/L, and the standard deviation at about 0.7 mmol/L. It was expected that triglyceride levels would drop to 1.5 mmol/L after CitriCholess intervention.

$$ N=\frac{2{\left({z}_a+{z}_B\right)}^2{\sigma}^2}{d^2} $$

σ:The estimated SD; d:The difference between two groups of continuous variables mean.

According to the two-sided test: α = 0.05 β = 0.1, Z_α_ **=**1.96 Z_β_ **=**1.282, the calculated sample size N is 41. Assuming a potential dropout rate of 20%, at least 48 people would be required for each group of the study. We enrolled 50 people each for the test group and control group.

### Randomization and blindness

The recruitment of the participants occurred at Tangqiao Community Health Service Center, Pudong New District, Shanghai, China. Volunteer participants were recruited by self-referral in response to media coverage and word of mouth. All study procedures were conducted in accordance with the Helsinki Declaration of 1975 and were approved by the Shanghai Jiao Tong University Institutional Review Board. Informed consent was obtained from all participants prior to enrollment into the study.

Subjects who met the inclusion criteria were eligible for the study: 1) Males or females subjects at least 50 years of age; 2) Fasting blood total triglyceride (TG) or total cholesterol (TC) concentrations higher than normal range (TG:0.56–1.70 mmol/L; TC: 2.8–5.7 mmol/L; HDL-C: 0.78–1.55 mmol/L; LDL-C: 1.68–4.53 mmol/L; glucose:3.9–6.1 mmol/L); Exclusion criteria: 1) Having diagnosed with any severe medical conditions or complications of the liver, kidneys, heart, lungs, or any other organs; 2) Having diagnosed with cancer; 3) having liver damage (such as serum glutamic-pyvuric transaminase (SGPT) or alanine aminotransferase (ALT) test and other anomalies); 4) Subjects who have much doubts of the study, or are unwilling or unlikely to keep adherence of the study procedure.

Participants were randomly assigned to the CitriCholess group or placebo group. The randomization was performed using a predetermined randomization code which was generated by a random number generator of the SPSS statistical software (Apendix-1).

Both trial participants and community doctors were blinded from the assignment (double-blind). Of the 100 enrolled participants, 98 participants completed the 12-week follow-up. Two subjects in the CitriCholess group withdrew from the study due to the symptom of dizziness.

### Medical history, physical examinations and blood analysis

A medical questionnaire that included the demographics, gender, race, history of alcohol consumption, current medical diagnosis, family medical history and medication history of the participants was evaluated before the start of the 12-week intervention. Blood concentrations of TG, TC, LDL-C, HDL-C and fasting blood glucose, and body weight (kg), waist and hip circumferences (cm), waist-to-hip- ratio and body mass index (BMI) were evaluated before and after the 12-week supplementation. All participants were followed up each month in order to check compliance and adverse effects.

### Supplementation procedure

All participants received similar-looking capsules in color-coded bottles (white bottles for CitriCholess and yellow bottles for placebo). Neither the subjects nor the medical doctors, including the study principal investigator, knew the specific color code until the end of the study. Both the CitriCholess capsules and the placebo, which was mainly composed of vegetable oil (soy oil), were manufactured and supplied by GardaVita Inc. (Costa Mesa, California, USA). Each participant was instructed to take 2 capsules with a meal, two times per day for 12 weeks and a new batch of supplements was dispensed every month during follow-up sessions. The key active ingredients of the CitriCholess formulation includes *Citrus bergamia* Risso extract (500 mg/daily) (25% bioflavonoids), plant sterol esters and orange oil (820 mg/daily), vitamin C (ascorbic acid) (50 mg/daily), Vitamin B6 (pyridoxine hydrochloride) (20 mg/daily), B12 (methylcobalamin) (2000 mcg/daily) and folic acid (800 mcg/daily).

### Statistics analysis

EpiData 3.02 software was used for the data entry and SPSS 20 software was used for statistical analysis. Group data were presented as the mean ± s.d. Differences between the CitriCholess and placebo groups were compared using Student’s t-test/1-way or 2-way ANOVA for quantitative variables with normal distribution, and Chi-square test were conducted for categorized variables. The alpha level of *P* > 0.05 was chosen as being statistically significant. All *p*-values reported were 2-sided.

## Results

### Demographic characteristics

The demographics and histories of alcohol intake, disease and medication of the participants are shown in Table 1. There were 17 males (35.4%) and 31 females (64.6%) in the CitriCholess group, and 11 males (22%) and 39 females (78%) in the placebo group. The gender distribution between the two groups was not significantly different between the two groups (χ^2^ = 2.16, *P* > 0.05), with 70 female participants accounting for 71.4% of all participants (98). The average age of all participants was 65.2 ± 9.1, and no significant difference was found between the CitriCholess group (66.7 ± 9.4 years) and the placebo group (63.8 ± 8.6 years) (*t* = 0.04, *P* > 0.05).

**Table 1:** Demographics and medical history of the participants (*N* = 98)

Items	Male N(%)			Female N(%)			Total N(%)			*χ* ^2^/ *P* value
	CitriCholess	Placebo	Combined	CitriCholess	Placebo	Combined	CitriCholess	Placebo	Combined	
N (% of subtotal)	17 (35.4)	11 (22.0)	28(28.6)	31 (64.6)	39 (78.0)	70(71.4)	48 (49)	50 (51)	98 (100)	2.16/ 0.142
Age, years (mean ± s.d)	65.5 ± 7.3	64.7 ± 10.0	65.2 ± 8.3	67.4 ± 10.4	63.6 ± 8.3	65.3 ± 9.4	66.7 ± 9.4	63.8 ± 8.6	65.2 ± 9.1	0.044/ 0.965
Age, years (range)	56.66–83.20	50.50–81.51	50.50–83.20	50.04–84.46	36.21–80.30	36.21–84.46	50.04–84.46	36.21–81.51	36.21–84.46	
Smoking (positive %)	4/17 (23.5)	5/11 (45.5)	9/28 (32.1)	0/31 (0.0)	0/39 (0.0)	0/70 (0.0)	4/48 (8.3)	5/50 (10.0)	9/98 (9.2)	0.082/ 0.775
Alcohol drinking (positive %)	5/17 (29.4)	1/11 (9.1)	6/28 (21.4)	0/31 (0.0)	0/39 (0.0)	0/70 (0.0)	5/48 (10.4)	1/50 (2.0)	6/98 (6.1)	1.732/ 0.188
High Blood Pressure (positive %)	8/17 (47.1)	5/11 (45.5)	13/28 (46.4)	23/31 (47.9)	25/39 (50)	48/70 (68.6)	31/48 (64..6)	30/50 (60.0)	61/98 (62.2)	0.336/ 0.953
Abnormal Glucose (positive %)	1 (2.1)	1 (2)	2/28 (7.1)	3 (6.2)	3 (6)	6/70 (8.6)	4/48 (8.3)	4/50 (8.0)	8/98 (8.2)	0.336/ 0.953
Hpertension& Abnormal Levels of Blood Glucose (positive %)	7 (14.6)	5 (10)	12/28 (42.9)	4 (8.3)	9 (18)	13/70 (18.6)	11/48 (22.9)	14/50 (28.0)	25/98 (25.5)	0.336/ 0.953
Antilipemic drug history (positive %)	1/17 (5.9)	2/11 (18.2)	3/28 (10.7)	2/31 (6.5)	3/39 (7.7)	5/70 (7.1)	3/48 (6.2)	5/50 (10.0)	8/98 (8.2)	0.031/ 0.861

The CitriCholess and placebo groups showed similar patterns of alcohol drinking (10.4% vs. 2.0%) (χ^2^ = 1.732, *P* = 0.188), smoking (8.3% vs. 10.0%) (χ^2^ = 0.082, *P* = 0.775), distribution of abnormal levels of blood glucose (8.3% vs. 8.0%%), high blood pressure (64.6% vs. 54.0%), abnormal levels of blood glucose and high blood pressure co-morbidity (22.9% vs. 28.0%%) (χ^2^ = 0.336, *P* = 0.953), and other health conditions (8.3% vs. 10.0%), history of anti-lipemic medication (6.3% vs. 10.0%) (χ^2^ = 0.095, *P* = 0.758) (Table 1). Only two people from each group were free from any chronic diseases.

All participants completed the 12-week follow-up observation except the 2 drop outs in the CitriCholess group due to the symptom of dizziness. There are no differences between the CitriCholess and Placebo groups in the adherence rate as reflected in the number of leftover pills after 1 month and 2 months follow-ups (Table 2).

**Table 2:** Leftover dosage after 1 and 2 months of the follow-up observation (n, %)

Leftover dosage	After 1 month			After 2 months		
	CitriCholess	Placebo	Subtotal	CitriCholess	Placebo	Subtotal
0 pill	12(25.5)	9(18.4)	21(21.9)	12(25.5)	9(18.4)	21(21.4)
1–10 pills	30(63.8)	37(75.5)	67(69.8)	11(22.9)	11(22.0)	22(22.4)
11–16 pills	5(10.6)	3(6.1)	8(8.3)	25(52.1)	30(60.0)	55(56.1)
Total	48(100.0)	50(100.0)	98(100.0)	48(100.0)	50(100.0)	98(100.0)
χ2 / *P*	1.626/ 0.443			0.843/ 0.656		

### Physical examination and blood analysis

*t*-test and 1 way-ANOVA showed no baseline differences between the CitriCholess and placebo groups in body weight (69.17 ± 12.65 kg vs. 68.88 ± 10.47 kg), waist circumference (86.42 ± 10.82 cm vs. 85.96 ± 9.56 cm), hip circumference (98.2 ± 6.7 cm vs. 98.8 ± 7.6 cm), waist-to-hip circumference ratio (0.879 ± 0.074 vs. 0.869 ± 0.573), BMI index (25.74 ± 3.23 vs. 26.27 ± 3.27), blood concentrations of cholesterol (5.46 ± 0.99 mmol/L vs. 5.62 ± 0.75 mmol/L), triglycerides (2.17 ± 0.81 mmol/L vs. 1.93 ± 0.54 mmol/L), HDL (1.29 ± 0.32 mmol/L vs. 1.34 ± 0.29 mmol/L), LDL (3.39 ± 0.91 mmol/L vs. 3.58 ± 0.74 mmol/L), and fasting glucose (6.16 ± 1.29 mmol/L vs. 6.48 ± 1.88 mmol/L) (*P* > .05, each) (Tables 3 and 4).

**Table 3:** Change in physical traits after 12-week CitriCholess intervention (mean ± s.d)

Items	CitriCholess	Placebo	t/*P* value
Height (cm)			
Before intervention	163.48 ± 8.13	161.76 ± 7.18	1.111/0.269
After intervention	163.42 ± 8.20	161.78 ± 7.15	1.055/0.294
Changed value	(−0.063) ± 0.433	0.020 ± 0.141	1.278/0.204
Weight (kg)			
Before intervention	69.17 ± 12.65	68.88 ± 10.47	0.122/0.903
After intervention	68.56 ± 12.41	68.94 ± 10.36	0.164/0.870
Changed value	(−0.604) ± 0.939	0.060 ± 0.740	3.878/<0.001**
BMI index			
Before intervention	25.74 ± 3.23	26.2 ± 3.27	0.818/0.415
After intervention	25.53 ± 3.10	26.30 ± 3.29	1.190/0.237
Changed value	(−0.207) ± 0.357	0.025 ± 0.274	3.599/0.001**
Waist circumference (cm)			
Before intervention	86.42 ± 10.82	85.96 ± 9.56	0.222/0.825
After intervention	85.81 ± 10.50	86.12 ± 9.33	0.153/0.878
Changed value	(−0.604) ± 1.349	(−0.160) ± 1.503	2.645/0.01**
Hip circumference (cm)			
Before intervention	98.2 ± 6.7	98.8 ± 7.6	0.451/0.653
After intervention	97.75 ± 6.505	98.62 ± 7.695	0.257/0.613
Changed value	(−0.396) ± 1.484	(−0.180) ± 1.289	0.770/0.443
Waist-to-hip circumference ratio			
Before intervention	0.879 ± 0.074	0.869 ± 0.573	0.737/0.463
After intervention	0.877 ± 0.072	0.873 ± 0.059	0.279/0.781
Changed value	(−0.0026) ± 0.0083	0.0036 ± 0.158	2.397/0.018*

*P < 0.05, differences between the CitriCholess and placebo groups after the 12-week supplementation

**P < 0.01, differences between the CitriCholess and placebo groups after the 12-week supplementation

**Table 4:** Change in blood cholesterol concentrations after 12-week CitriCholess intervention (mean ± s.d)

Items	CitriCholess	Placebo	t / *P* value
Cholesterol(mmol/L)			
Before intervention	5.46 ± 0.99	5.62 ± 0.75	0.895/ 0.373
After intervention	5.14 ± 0.98	5.44 ± 0.77	1.677/ 0.097
Changed value	(−0.32) ± 0.86	(−0.18) ± 0.77	0.851/ 0.397
Triglyceride(mmol/L)			
Before intervention	2.17 ± 0.81	1.93 ± 0.54	1.760/ 0.082
After intervention	1.83 ± 0.92	1.95 ± 1.34	0.509/ 0.612
Changed value	(−0.347) ± 0.916	0.02 ± 1.309	1.589/ 0.115
HDL(mmol/L)			
Before intervention	1.29 ± 0.32	1.34 ± 0.29	0.862/ 0.391
After intervention	1.30 ± 0.27	1.35 ± 0.28	0.861/ 0.391
Changed value	0.0081 ± 0.235	0.0034 ± 0.202	0.107/ 0.915
LDL(mmol/L)			
Before intervention	3.39 ± 0.91	3.58 ± 0.74	1.089/ 0.279
After intervention	3.13 ± 0.74	3.43 ± 0.62	2.17/ 0.032*
Changed value	(−0.269) ± 0.750	(−0.151) ± 0.620	0.846/ 0.400
Glucose(mmol/L)			
Before intervention	6.16 ± 1.29	6.48 ± 1.88	0.966/ 0.336
After intervention	6.06 ± 1.49	6.63 ± 2.13	1.547/ 0.125
Changed value	(−0.104) ± 1.090	0.156 ± 0.971	1.249/ 0.215

*P < 0.05, differences between the CitriCholess and placebo groups after the 12-week supplementation

After the 12 weeks of CitriCholess supplementation, the CitriCholess group showed non-significant reductions (*P* < 0.05) whereas the placebo group showed a non-significant increases (*P* < 0.05) in body weight, BMI, waist and hip circumferences, and waist-to-hip ratio. The absolute value change were significant in body weight, waist circumference, waist-to-hip circumference ratio and BMI between the two groups (*P* < 0.05, each).

The CitriCholess group also showed non-significant reductions in blood concentrations of TC, TG, LDL and fasting glucose whereas the placebo group showed a non-significant reduction in TC and LDL and a slight increase in TG and fasting glucose level after the 12-week CitriCholess supplementation (Table 4). Thus, blood LDL levels became significantly lower in the CitriCholess group (3.13 ± 0.74 mmol/L) than in the placebo group (3.43 ± 0.62 mmol/L) (*P* < 0.05) after the 12-week supplementation.

Two-way ANOVA were also conducted to determine the effects and potential interactions of supplementation and gender. As expected, the males had significantly greater mean baseline values than females in body weight (79.00 ± 10.60 vs. 65.03 ± 9.29 kg) (*P* < 0.01), height (171.43 ± 5.59 vs. 159.07 ± 5.12 cm) (*P* < 0.01), waist circumference (91.64 ± 9.99 vs. 84.0 ± 9.42 cm) (*P* < 0.01), and fasting glucose (6.95 ± 2.32 vs. 6.07 ± 1.16 mmol/L) (*P* < 0.01), but significantly lower values in HDL (1.15 ± 0.28 vs.1.39 ± 0.30 mmol/L) (*P* < 0.01) (Tables 5 and 6). These significant gender differences remained unchanged after the 12-weekCitriCholess supplements.

**Table 5:** Sex difference in physical examination before and after the 12-week intervention (mean ± s.d)

Items	Male		Female	
	Before intervention	After intervention	Before intervention	After intervention
Height (cm)				
CitriCholess	171.06 ± 6.15	171.06 ± 6.15	159.32 ± 5.75	159.23 ± 5.81
Placebo	172.00 ± 4.84	172.00 ± 4.84	158.87 ± 4.62	158.90 ± 4.58
t / *P* value	0.428/ 0.672	0.428/ 0.672	0.364/ 0.717	0.264/ 0.792
Weight (kg)				
CitriCholess	79.18 ± 12,20	78.24 ± 12.19	63.68 ± 9.13	63.26 ± 8.94
Placebo	78.73 ± 8.09	78.45 ± 8.07	66.10 ± 9.38	66.26 ± 9.37
t / *P* value	0.107/ 0.915	(−0.053) / 0.959	(−1.087)/0.281	(−1.357)/0.179
BMI index				
CitriCholess	27.00 ± 3.39	26.67 ± 3.33	25.04 ± 2.96	24.90 ± 2.83
Placebo	26.64 ± 2.87	26.55 ± 2.83	26.16 ± 3.40	26.22 ± 3.43
t / *P* value	0.289/ 0.775	0.098/ 0.923	(−1.458) / 0.150	(−1.729)/0.088
Waist circumference (cm)				
CitriCholess	93.00 ± 11.62	92.35 ± 11.47	82.81 ± 8.56	82.23 ± 8.07
Placebo	89.55 ± 6.74	90.00 ± 5.42	84.95 ± 10.05	85.03 ± 9.50
t / *P* value	0.994/ 0.329	0.663/ 0.532	(−0.945) / 0.348	(−1.270) / 0.209
Hip circumference (cm)				
CitriCholess	100.35 ± 5.93	100.18 ± 6.17	96.94 6.87	96.42 ± 6.39
Placebo	100.82 ± 5.46	100.00 ± 6.21	98.23 8.09	98.23 ± 8.09
t / *P* value	(−0.209)/ 0.836	0.074/ 0.942	(−0.711)/ 0.480	(−1.019)/ 0.312
Waist-to-hip circumference ratio				
CitriCholess	0.925 ± 0.084	0.920 ± 0.079	0.854 ± 0.055	0.852 ± 0.056
Placebo	0.889 ± 0.060	0.902 ± 0.065	0.864 ± 0.056	0.865 ± 0.055
t / *P* value	1.244/ 0.225	0.632/ 0.533	(−0.734)/ 0.466	(−0.899)/ 0.372

**Table 6:** Sex difference in blood metabolites before and after the 12-week intervention (mean ± s.d)

Items	Male	Female		
	Before intervention	After intervention	Before intervention	After intervention
Cholesterol(mmol/L)				
CitriCholess	5.53 ± 1.25	5.06 ± 0.96	5.42 ± 0.83	5.18 ± 1.00
Placebo	5.45 ± 0.90	5.38 ± 0.58	5.67 ± 0.71	5.46 ± 0.82
t / *P* value	0.185/ 0.855	(−1.081) / 0.290	(−1.328) / 0.189	(−1.249) / 0.216
Triglyceride(mmol/L)				
CitriCholess	2.38 ± 0.85	1.85 ± 0.73	2.06 ± 0.78	1.82 ± 1.02
Placebo	2.04 ± 0.37	2.02 ± 0.71	1.90 ± 0.58	1.93 ± 1.48
t / *P* value	1.258/ 0.219	(−0.595) / 0.557	1.006/ 0.318	(−0.357) / 0.722
HDL(mmol/L)				
CitriCholess	1.13 ± 0.28	1.18 ± 0.20	1.38 ± 0.29	1.36 ± 0.29
Placebo	1.19 ± 0.29	1.18 ± 0.19	1.39 ± 0.28	1.40 ± 0.28
t / *P* value	(−0.580) / 0.567	0.079/ 0.938	(−0.079) / 0.937	(−0.468) / 0.641
LDL(mmol /L)				
CitriCholess	3.33 ± 1.02	3.32 ± 0.75	3.43 ± 0.86	3.01 ± 0.73
Placebo	3.54 ± 0.87	3.46 ± 0.50	3.59 ± 0.71	3.42 ± 0.66
t / *P* value	(−0.546) / 0.590	(−0.509) / 0.615	(−0.847) / 0.400	(−2.419) / 0.018*
Glucose(mmol/L)				
CitriCholess	6.72 ± 1.61	6.53 ± 2.13	5.86 ± 0.97	5.80 ± 0.92
Placebo	7.34 ± 3.19	7.46 ± 3.63	6.24 ± 1.27	6.40 ± 1.45
t / *P* value	(−0.564) / 0.582	(−0.861) / 0.397	(−1.408) / 0.164	(−2.103) / 0.039*

*P < 0.05, differences between the CitriCholess and placebo groups after the 12-week supplementation

Co-variance analysis, adjusted on body weight, show significant effects of gender on baseline HDL (*P* = 0.015) and trend effect of gender on fasting glucose (*P* = 0.067) with the males had lower HDL but higher fasting glucose than the females. In addition, co-variance analysis of the post 12-week supplementation data, adjusted with body weight, show greater lowering effects by CitriCholess than by placebo on cholesterol (5.154 ± 0.134 mmol/L vs. 5.472 ± 0.154 mmol/L) (*P* = 0.116), LDL (3.193 ± 0.103 mmol/L vs. 3.475 ± 0.119 mmol/L) (*P* = 0.072) and glucose (6.156 ± 0.278 mmol/L vs. 6.916 ± 0.319 mmol/L) (*P* = 0.072).

No significant differences were found between the CitriCholess and placebo groups in the distribution pattern of the participants with normal and abnormal blood profile before or after the 12-week supplementation (Tables 7, 8, 9, 10 and 11).

**Table 7:** Before x After 12-week treatment cross-tabulate: Triglyceride (TG) (n, %)

Group	TG before treatment	TG after treatment		
		Normal	Abnormal	Total
CitriCholess s	Normal	7(77.8)	2(22.2)	9(18.8)
	Abnormal	18(46.2)	21(53.8)	39(81.3)
	Subtotal	25(52.1)	23(47.9)	48(100.0)
Placebo	Normal	12(75.0)	4(25.0)	16(32.0)
	Abnormal	14(41.2)	20(58.8)	34(68.0)
	Subtotal	26(52.0)	24(48.0)	50(100.0)

**Table 8:** Before x After 12-week treatment cross-tabulate: Cholesterol (TC) (n, %)

Group	TC before treatment	TC after treatment		
		Normal	Abnormal	Total
CitriCholess s	Normal	25(92.6)	2(7.40)	27(56.25)
	Abnormal	9(42.9)	12(57.1)	21(43.75)
	Subtotal	34(70.8)	14(29.2)	48(100.0)
Placebo	Normal	23(95.8)	1(4.2)	24(48.0)
	Abnormal	9(34.6)	17(65.4)	26(52.0)
	Subtotal	32(64.0)	18(36.0)	50(100.0)

**Table 9:** Before x After 12-week treatment cross-tabulate: LDL-C (n, %)

Group	LDL-C before treatment	LDL-C after treatment		
		Normal	Abnormal	Total
CitriCholess s	Normal	40(95.2)	2(4.80)	42(87.5)
	Abnormal	5(83.3)	1(16.7)	6(12.5)
	Subtotal	45(93.8)	3(6.2)	48(100.0)
Placebo	Normal	45(97.8)	1(2.2)	46(92.0)
	Abnormal	3(75.0)	1(25.0)	4(8.0)
	Subtotal	48(96.0)	2(4.0)	50(100.0)

**Table 10:** Before x After 12-week treatment cross-tabulate: HDL-C (n, %)

Group	HDL-C before treatment	HDL-C after treatment		
		Normal	Abnormal	Total
CitriCholess s	Normal	37(97.4)	1(2.6)	38(79.17)
	Abnormal	3(30.0)	7(70.0)	10(20.83)
	Subtotal	40(83.3)	8(16.7)	48(100.0)
Placebo	Normal	40(95.2)	2(4.8)	42(84.0)
	Abnormal	2(25.0)	6(75.0)	8(16.0)
	Subtotal	42(84.0)	8(16.0)	50(100.0)

**Table 11:** Before x After 12-week treatment cross-tabulate: Glucose (n,%)

Group	Glucose before treatment	Glucose after treatment		
		Normal	Abnormal	Total
CitriCholess s	Normal	29(96.7)	1(3.3)	30(62.5)
	Abnormal	5(27.8)	13(72.2)	18(37.5)
	Subtotal	34(70.8)	14(29.2)	48(100)
Placebo	Normal	28(93.3)	2(6.7)	30(60.0)
	Abnormal	1(5.0)	19(95.0)	20(40.0)
	Subtotal	29(58.0)	21(42.0)	50(100.0)

Cross-tabulation of the distribution of the TG showed that 18 of the 39 (46.2%) people with abnormal baseline blood TG level in the CitriCholess group showed normal blood TG level after 12-week CitriCholess supplementation, whereas 14 out of the 34 people (41.2%) with abnormal baseline TG level in the placebo group showed normal blood TG level after 12 weeks placebo treatment (Table 7). Additionally, 2 of the 9 people (22.2%) in the CitriCholess group and 4 of the 16 people (25%) in the placebo group with normal baseline blood TG level showed abnormal blood TG level after the 12-week treatment. Thus there were bi-directional changes between the two groups.

Similarly, 9 out of the 21 (42.9%) people in the CitriCholess group and 9 out of the 26 people (34.6%) in the placebo group with abnormal baseline blood TC level showed normal blood TG level after the 12-week treatment (Table 8). Additionally, 2 of the 27 people (7.4%) in the CitriCholess group and 1 of the 24 people (4.2%) in the placebo group with normal baseline blood TG level showed abnormal blood TC level after the 12-week program.

One out of the 6 (16.7%) people in the CitriCholess group and 1 out of the 4 people (25%) in the placebo group with abnormal baseline blood LDL-C level showed normal blood LDL-C level after the 12-week treatment (Table 9). Additionally, 2 of the 40 people (4.8%) in the CitriCholess group and 1 of the 45 people (2.2%) in the placebo group with normal baseline blood LDL-C level showed abnormal blood LDL-C level after the 12-week program.

Seven out of the 10 (70.0%) people in the CitriCholess group and 6 out of the 8 people (75%) in the placebo group with abnormal baseline blood HDL-C level showed normal blood HDL-C level after the 12-week treatment (Table 10). Additionally, 1 of the 38 people (2.6%) in the CitriCholess group and 2 of the 42 people (4.8%) in the placebo group with normal baseline blood HDL-C level showed abnormal blood HDL-C level after the 12-week program.

Similarly, 13 out of the 18 (72.2%) people in the CitriCholess group and 19 out of the 20 people (95%) in the placebo group with abnormal baseline blood glucose level showed normal blood glucose level after the 12-week treatment (Table 11). Additionally, 1 of the 30 people (3.3%) in the CitriCholess group and 2 of the 30 people (6.7%) in the placebo group with normal baseline blood glucose level showed abnormal blood glucose level after the 12-week treatment.

Correlations between physical traits and blood chemistry were also determined for the baseline values of the pooled and the CitriCholess and placebo groups (Tables 12, 13 and 14) and for the after treatment values of the pooled and the CitriCholess and placebo groups (Tables 15, 16 and 17). Generally, cholesterol is significant correlated with LDL-C regardless of gender, time and treatment, and to a less degree, correlated with TG and HDL-C. TG was negatively correlated with HDL-C and LDL-C but positively correlated with glucose. Body weight and waist circumference were negatively correlated with HDL-C and positively correlated with fasting glucose.

**Table 12:** Baseline correlations for the pooled samples (*N* = 98)

Items	Age	Weight	Height	Waist	Hip	TC	TG	HDL	LDL	Glucose
Age	1									
Weight	−0.129	1								
Height	−0.183	0.666^**^	1							
Waist	0.019	0.813^**^	0.358^**^	1						
Hip	0.039	0.717^**^	0.359^**^	0.769^**^	1					
TC	−0.084	−0.093	−0.034	−0.03	−0.082	1				
TG	0.127	0.159	0.112	0.229^*^	0.068	−0.008	1			
HDL	−0.058	−0.253^*^	−0.237^*^	−0.248^*^	−0.141	0.390^**^	−0.405^**^	1		
LDL	−0.056	−0.103	−0.082	−0.079	−0.048	0.674^**^	−0.383^**^	0.292^**^	1	
Glucose	−0.011	0.204^*^	0.163	0.305^**^	0.156	−0.022	0.16	−0.132	−0.141	1

^*^Significant Pearson correlation (*P* < 0.05 at a bilateral level)

^**^Significant Pearson correlation (*p* < 0.01 at a bilateral level)

**Table 13:** Baseline correlations within the CitriCholess group (*N* = 48)

Items	Age	Weight	Height	Waist	Hip	TC	TG	HDL	LDL	Glucose
Age	1									
Weight	−0.343^*^	1								
Height	−0.378^**^	0.731^**^	1							
Waist	−0.126	0.843^**^	0.481^**^	1						
Hip	−0.13	0.741^**^	0.467^**^	0.741^**^	1					
TC	0.048	0.035	0.012	0.169	0.082	1				
TG	0.152	0.027	0.012	0.139	−0.038	0.163	1			
HDL	−0.054	−0.27	−0.19	−0.239	−0.068	0.348^*^	−0.420^**^	1		
LDL	0.119	−0.023	−0.068	−0.001	0.058	0.582^**^	−0.389^**^	0.397^**^	1	
Glucose	0.036	0.248	0.118	.378^**^	0.174	−0.021	0.223	−0.25	−0.251	1

^*^Significant Pearson correlation (*P* < 0.05 at a bilateral level)

^**^Significant Pearson correlation (*p* < 0.01 at a bilateral level)

**Table 14:** Baseline correlations within the placebo group(N = 50)

Items	Age	Weight	Height	Waist	Hip	TC	TG	HDL	LDL	Glucose
Age	1									
Weight	0.132	1								
Height	−0.002	0.588^**^	1							
Waist	0.183	0.774^**^	0.205	1						
Hip	0.21	0.713^**^	0.276	0.812^**^	1					
TC	−0.235	−0.287^*^	−0.075	−0.312^*^	−0.278	1				
TG	0.025	0.394^**^	0.23	0.380^**^	0.23	−0.289^*^	1			
HDL	−0.035	−0.233	−0.275	−0.257	−0.218	0.441^b^	−0.376^**^	1		
LDL	−0.241	−0.216	−0.073	−0.179	−0.17	0.809^b^	−0.343^*^	0.146	1	
Glucose	−0.017	0.188	0.23	0.274	0.141	−0.043	0.168	−0.071	−0.085	1

^*^Significant Pearson correlation (*P* < 0.05 at a bilateral level)

^**^Significant Pearson correlation (*p* < 0.01 at a bilateral level)

**Table 15:** After 12-week treatment correlations for the pooled samples (*N* = 98)

Items	Age	Weight	Height	Waist	Hip	TC	TG	HDL	LDL	Glucose
Age	1									
Weight	−0.121	1								
Height	−0.182	0.663^**^	1							
Waist	0.048	0.807^**^	0.370^**^	1						
Hip	0.055	0.699^b^	0.345^**^	0.758^**^	1					
TC	0.065	−0.165	−0.125	−0.086	−0.055	1				
TG	0.135	0.006	−0.004	0.157	0.01	0.316^**^	1			
HDL	0.012	−0.264^**^	-.232^*^	−0.336^**^	−0.159	0.274^**^	−0.326^**^	1		
LDL	0.018	−0.039	0.025	−0.004	0.049	0.700^**^	−0.167	0.278^**^	1	
Glucose	−0.008	0.135	0.16	0.256^*^	0.133	0.181	0.356^**^	−0.202^*^	0.07	1

^*^Significant Pearson correlation (*P* < 0.05 at a bilateral level)

^**^Significant Pearson correlation (*p* < 0.01 at a bilateral level)

**Table 16:** After 12-week treatment correlations witin the CitriCholess group (*N* = 48)

Items	Age	Weight	Height	Waist	Hip	TC	TG	HDL	LDL	Glucose
Age	1									
Weight	−0.320^*^	1								
Height	−0.372^**^	0.744^**^	1							
Waist	−0.091	0.826^**^	0.480^**^	1						
Hip	−0.073	0.723^**^	0.454^**^	0.743^**^	1					
TC	0.206	−0.105	−0.132	0.033	0.076	1				
TG	0.239	0.096	0.066	0.287^*^	0.117	0.176	1			
HDL	0.086	−0.2	−0.188	−0.28	−0.087	0.251	−0.377^*^	1		
LDL	0.205	0.021	0.073	0.13	0.135	0.739^**^	−0.063	0.295^*^	1	
Glucose	0.014	0.125	0.21	0.257	0.08	0.065	0.203	−0.27	0.123	1

^*^Significant Pearson correlation (*P* < 0.05 at a bilateral level)

^**^Significant Pearson correlation (*p* < 0.01 at a bilateral level)

**Table 17:** After 12-week treatment correlations within the Placebo group (*N* = 50)

Items	Age	Weight	Height	Waist	Hip	TC	TG	HDL	LDL	Glucose
Age	1									
Weight	0.129	1								
Height	−0.001	0.570^**^	1							
Waist	0.217	0.784^**^	0.241	1						
Hip	0.188	0.697^**^	0.27	0.788^**^	1					
TC	−0.052	−0.267	−0.077	−0.258	−0.215	1				
TG	0.084	−0.066	−0.048	0.068	−0.055	0.452^**^	1			
HDL	−0.036	−0.344^*^	−0.267	−0.403^**^	−0.228	0.284^*^	-.312^*^	1		
LDL	−0.133	−0.132	0.018	−0.186	−0.057	0.619^**^	-.289^*^	0.236	1	
Glucose	0.02	0.149	0.166	0.268	0.151	0.252	0.421^**^	−0.19	−0.029	1

^*^Significant Pearson correlation (*P* < 0.05 at a bilateral level)

^**^Significant Pearson correlation (*p* < 0.01 at a bilateral level)

## Discussion

Overweight, abnormal cholesterol levels, higher-than-normal blood pressures and elevated fasting glucose can synergistically and independently predispose people to increased cardiovascular dysfunction [44–47]. Currently, statins are the primary lipid-modifying drugs for people with an abnormal cholesterol levels. Despite optimal statin therapy, however, many people fail to achieve therapeutic goals with statin monotherapy. Approximately 10% to 20% of people develop statin intolerance especially at high doses of statins [14, 48], and discontinuation of statin intervention is common and many people still had CVD events after statin treatment [49–51]. Thus alternative approaches including dietary supplements and nutraceuticals are needed.

In this study, supplementation of CitriCholess for 12 weeks appeared to have reduced blood concentrations of cholesterol, triglycerides, LDL-C, glucose, waist circumference, body weight, and BMI in old adults with dyslipidemia than placebo although some of the improvement did not reach a significant level. This may reflect the fact that the number of the participants was relatively small and some of them had normal or near-normal baseline values at the start of the study. In addition, the relatively small daily dose of CitriCholess and short supplement duration as well as the medical treatment for blood glucose and blood pressure issues of the participants, and the enhanced awareness of lifestyle modification could not be ignored entirely.

Nevertheless, the generally greater reductions in blood concentrations of cholesterol, triglycerides, LDL-C, glucose, waist circumference, body weight, and BMI in the CitriCholess group than in the placebo group support a beneficial effect of CitriCholess supplementation in old adults.

Our observed greater reductions in body weight and BMI in the CitriCholess group are in agreement with the recent report that oral administration of Bergamot polyphenolic fraction (BPF) (1000 mg/day) for 30 days resulted in significant reductions of body weight (*P* < 0.005) and BMI (*P* = 0.005) in 15 subjects with metabolic syndrome (due to atypical antipsychotics treatment) [3]. Interestingly, that study did not find significant changes in fasting levels of blood glucose, glycated hemoglobin, total cholesterol, HDL-C, LDL-C and triglycerides probably after a relatively short 30 days of observation [3].

In fact, in another recent study of 80 subjects with moderate high cholesterol levels (plasma LDL-C between 4.1 and 4.9 mmol/l), bergamot extract (flavonoids, BEF) (150 mg/day, 16% of neoeriocitrin, 47% of neohesperidin and 37% of naringin) supplement for 6 months significantly reduced total cholesterol from 6.6 to 5.8 mmol/L (*p* < 0.0001), reduced triglycerides from 1.8 to 1.5 mmol/L (*p* < 0.01), and LDL-C from 4.6 to 3.7 mmol/L (p < 0.0001), while HDL-C increased from 1.3 to 1.4 mmol/L (*p* < 0.001) [52].

Babish et al. [53] also reported that 12 weeks of F105 supplementation, a *Citrus bergamia*-based herbal formulation, significantly reduced total cholesterol (−7.3%), LDL-C (−10%), non-HDL-C (−7.1%), cholesterol/HDL ratio (−26%), and apolipoprotein B (−2.8%) in 11 subjects with moderate-abnormal cholesterol levels. Subgroup analysis of 8 subjects with HbA1c > 5.4 or elevated triglycerides showed even greater reductions in triglycerides (−27%), oxLDL (−19%), LDL/HDL (−25%), triglycerides/HDL (−27%), oxLDL/HDL (−25%), and PAI-1 (−37%) than the group average. Further, a 70-year-old female previously not nonresponsive to statin therapy showed significant improvement after F105 supplementation.

Gliozzi et al. [54] compared the vasoprotective and lipid-lowering efficacies of bergamot polyphenolic fraction (BPF) with that of statins in a randomized placebo-controlled study of 77 subjects with abnormal cholesterol levels who were divided into 5 subgroups: 1) placebo (*n* = 15); 2) two groups of oral rosuvastatin (10 and 20 mg/daily for 30 days; *n* = 16, each group); 3) BPF alone group (1000 mg/daily for 30 days; n = 15); 4) BPF (1000 mg/daily) plus rosuvastatin group (10 mg/daily for 30 days; n = 15). Compared with placebo, both doses of rosuvastatin (10 or 20 mg) and BPF significantly reduced TC (195, 174 and 191 mg/dl, respectively, vs. 275 mg/dl of placebo, *p* < 0.05), LDL-C (115, 87 and 113 mg/dl, respectively, vs. 190 mg/dl of placebo, p < 0.05). Bergamot plus rosuvastatin significantly enhanced the suppressing effect of rosuvastatin on TG levels (152 mg/dl (1000 mg BPF + 10 mg rosuvastatin, and 200 mg/dl (10 mg rosuvastatin), respectively, vs. 235 mg/dl of placebo, *p* < 0.05). Moreover, the enhanced lipid-lowering effect of BPF was associated with significant reductions in biomarkers of vascular oxidative damage (malondialdehyde, oxyLDL receptor LOX-1 and protein kinase B) [54].

In a double-blind, randomized, placebo-controlled trial, Mollace et al. [34]compared the effects of bergamot extract (BE) treatments with that placebo controls in 237 people with 4 different subtypes of abnormal cholesterol levels: 104 with high cholesterol levels (HC) (500 mg/day BE); 2) 42 with high levels of fats in the blood (high cholesterol levels and high triglycerides levels, HC/HT) (1000 mg/day BE); 3) 59 with high levels of fats in the blood plus high blood sugar (MetS group, HC/HT/HG); and 4) 32 with abnormal cholesterol levels who discontinued simvastatin treatment due to adverse effects (1500 mg/day BE). Oral bergamot (500 mg/day or 1000 mg/day) significantly decreased TC (21.8%, and 29.4%, respectively, vs. placebo; *p* < 0.001, for each dose), LDL-C (24.1% and 30.6%, respectively, vs. placebo; *p* < 0.001, for each dose), and dose-dependently increased HDL-C levels in most subjects with high cholesterol levels and/or high triglyceride levels/high blood sugar (22.3%, and 40.1%, respectively, vs. placebo; p < 0.001 for each dose). A significant reduction in TG levels in subjects with high triglyceride levels (28.2% for 500 mg/day, 37.9% for 1000 mg/day vs. placebo; p < 0.001 for each dose), and improvement in the glycemic profile in subjects with high blood sugar (18.9% for 500 mg/day, 22.4% for 1000 mg/day vs. placebo (*p* < 0.0001) were also observed. Furthermore, bergamot treatment (1500 mg/day) of those who discontinued statins significantly decreased TC and LDL-C levels (by 25 and 27.6%, respectively; *p* < 0.001 each) without side effects. Thus the bergamot supplement improved both lipid and glucose metabolism in subjects with different subtypes of abnormal cholesterol levels and statins resistance [34].

The mechanisms underlying the effects of lowering levels of both cholesterol and lipids/fats in the blood of C. bergamot remain to be fully understood. The buteridine, naringin and melitidine of BPF are structurally similar to HMG-CoA reductase substrate statins and thus can competitively inhibit HMG-CoA reductase [55] and reduce cholesterol and mevalonate levels [54]. BPF flavonoids may also lower cholesterol levels by binding bile acids and increasing the turnover rate of blood and liver cholesterol, reducing TG accumulation in the liver, and enhancing the excretion of fecal neutral sterols and total bile acids [32, 56]. Naringin and neohesperidin may reduce hepatic TG accumulation by inhibiting the activity of phosphatidate phosphohydrolase, a TG synthetic enzymes and by reducing the availability of lipids for assembly of lipoproteins via reduced activities of acyl CoA:cholesterol acyltransferases [57, 58] [59`, 60]. BPF flavonoids low blood sugar effects may be achieved by increasing AMP kinase activity and glucose uptake in muscle cells and liver [2] and by increased insulin sensitivity and glucose tolerance [59].

### Limitations

There are limitations and shortcomings of this study. The results were based on small number of participants with mild to moderate abnormal cholesterol levels whose inclusion criteria was not very strictly observed. In addition, the short duration of the study and a relatively low dose of BF present in the CitriCholess formulation (compared to other reported more effective studies) may have disallowed the detection of a maximal effect to be observed in old subjects with metabolic symptom and/or high blood-pressure.

## Conclusion

CitriCholess supplement for 12 week was safe and beneficial on body weight control, BMI and lipid profile in old adults with blood pressure and blood glucose issues.

## Abbreviations

BMI: Body weight index
BPF: Bergamot polyphenolic fraction
CBF: *Citrus bergamia* juice-derived flavonoids
CitriCholess: A dietary supplement
CVD: Cardiovascular conditions
HDL-C: High density lipoprotein cholesterol
HMG-CoA: (3-hydroxy- 3-methylglutaryl Co-enzyme A
LDL-C: Low density lipoprotein cholesterol
n3-PUFA: Omega-3 poly-unsaturated fatty acids
PAI-1: Plasminogen activator inhibitor-**1**
SBP: Systolic blood pressure
SGPT: Test of serum glutamic-pyvuric transaminase, or alanine aminotransferase (ALT) test for screening liver disease
SNPs: Single nucleotide polymorphisms
TC: Total cholesterol
